# Moment of induction and duration of experimental varicocele in rats: effects on semen quality

**DOI:** 10.1590/S1677-5538.IBJU.2023.0412

**Published:** 2024-04-25

**Authors:** Renata Cristina de Carvalho, Rhayza Roberta Andretta, Jheysson Alfredo Cordeiro de Moura, Ricardo Pimenta Bertolla, Fatima Kazue Okada

**Affiliations:** 1 Divisão de Urologia Departamento de Cirurgia Universidade Federal de São Paulo São Paulo SP Brasil Seção de Reprodução Humana, Divisão de Urologia, Departamento de Cirurgia, Universidade Federal de São Paulo, São Paulo, SP, Brasil;; 2 Laboratório de Biologia do Desenvolvimento Departamento de Morfologia e Genética Universidade Federal de São Paulo São Paulo SP Brasil Laboratório de Biologia do Desenvolvimento, Departamento de Morfologia e Genética, Universidade Federal de São Paulo, São Paulo, SP, Brasil

**Keywords:** Spermatozoa, Acrosome, Genitalia

## Abstract

**Purpose:**

Varicocele is a condition known to cause damage to seminal parameters and sperm function. Furthermore, it has been hypothesized that the varicocele effect on fertility is time-dependent; however, little is known about the consequences of its establishment time on reproductive organs and/or sperm function. This study aimed to evaluate the effect of the duration of experimental varicocele on reproductive organs, sperm parameters, and sperm function.

**Materials and Methods:**

Varicocele induction surgeries were performed in Wistar rats aged 40 or 100 days old. At 160-day-old, analyses were performed, including biometry of reproductive organs (prostate, seminal vesicles, epididymis, and testis), sperm parameters (vitality, morphology, and motility), and sperm function tests (nuclear DNA integrity, acrosome integrity, and mitochondrial activity).

**Results:**

The analysis of the biometry of reproductive organs showed no differences between distinct ages in which varicocele was induced. The total abnormal sperm morphology was bigger in animals with varicocele induced to 100 days old than in animals with varicocele induced to 40 days old. Regarding nuclear DNA integrity, animals of varicocele induced to 100 days old showed worse results compared to animals of varicocele induced to 40 days old. Other parameters analyzed showed no differences between varicocele groups.

**Conclusion:**

In this study conducted on rats, we conclude that varicocele adversely affects sperm, particularly its function. However, we did not observe a negative progressive effect on sperm.

## INTRODUCTION

Varicocele is defined as a pampiniform plexus vein dilation with retrograde blood flow in the spermatic vein. It is considered the main treatable cause of male infertility (1, 2) and its pathophysiology involves several mechanisms that can disturb the male fertility potential. Therefore, studies have demonstrated that venous dilatation increases the scrotal temperature, and leads to the occurrence of venous reflux, venous stasis, hypoxia, and oxidative stress (3).

Varicocele is detected in 15% of the adult male population (3). This disease affects 35% of men with primary infertility and 80% of those who present secondary infertility (4); researchers have believed that the damage caused by varicocele on fertility occurs progressively; however, there are no publications that corroborate this fact or show the relation of the establishment time of varicocele and male fertility aspects, such as sperm function (5). When analyzing adolescents with varicocele, an increase in the percentage was observed, which reaches more than 25% (6, 7).

Although studies of varicocele in humans are conducted, the establishment time of varicocele is too complicated to define in this species (8) because most men only discover varicocele when they have difficulty getting a child (9). On the other hand, studies performed on rats allow for knowing exactly when the varicocele was installed; furthermore, it is possible to study its tissue (10, 11) and its contralateral effect. This is considered a well-established model because it corroborates fertility alterations in humans, such as the decrease in sperm motility and the increase in sperm DNA damage (12).

Therefore, we hypothesize that the duration of varicocele affects progressively semen quality. Thereby, we purposed to verify if varicocele progressively impacts male fertility. Thus, this study aimed to understand the effects of the duration of experimental varicocele on reproductive organs, sperm parameters, and sperm function.

## MATERIALS AND METHODS

The experiments were conducted according to the rules issued by the National Council for Control of Animal Experimentation and approved by the Local Ethics Committee of Animal 7075090516.

### Animals and Groups

Eighteen male Wistar rats (*Rattus norvegicus albinus*) were distributed in three groups: Varicocele 40 (V40, n=6), varicocele induction surgery at 40-day-old; Varicocele 100 (V100, n=6), varicocele induction surgery at 100-day-old; Sham-control (S, n=6), surgery without varicocele induction.

All animals were housed in individually ventilated cages with a controlled environment (12/12h light/dark 23 °C-25 °C), water, and food were provided *ad libitum.*

The experiments were conducted under the Guide for the Care and Use of Laboratory Animals of National Institutes of Health.

### Varicocele induction surgery

The surgery was conducted according to the method previously described (8, 11). Rats were anesthetized with Ketamine (75 mg/Kg of body weight, Cetamin, Syntec-Brazil) and Xylazine (9 mg/kg of body weight, Anasedan, Ceva-Brazil) intraperitoneally. All steps of varicocele induction are described in Okada *et al.* (11).

Animals of the Sham-control group were submitted to the same conditions as the animals from Varicocele groups, including the surgery, but without the varicocele induction. In this way, even the discomfort and distress of the procedure were considered.

For analgesia, Meloxicam (1 mg/kg of body weight, Maxicam, Ourofino-Brazil) was administered at the beginning of the surgery and for 5 consecutive days afterward.

At 160-day-age, rats were euthanized with an intraperitoneal injection of Thiopental sodium (120 mg/kg of body weight, Cristalia-Brazil).

### Morphometric procedure and sperm recovery

Testis, prostate, epididymis, and seminal vesicles were collected and weighed. Then, the volume of the testes was measured by Scherle’s method (13).

The epididymis cauda was isolated to obtain sperm. Each epididymis was cut and submerged in Biggers Whitten Whittingan medium at a temperature of 37°C for 15 minutes.

### Vitality

**Figure 1:**
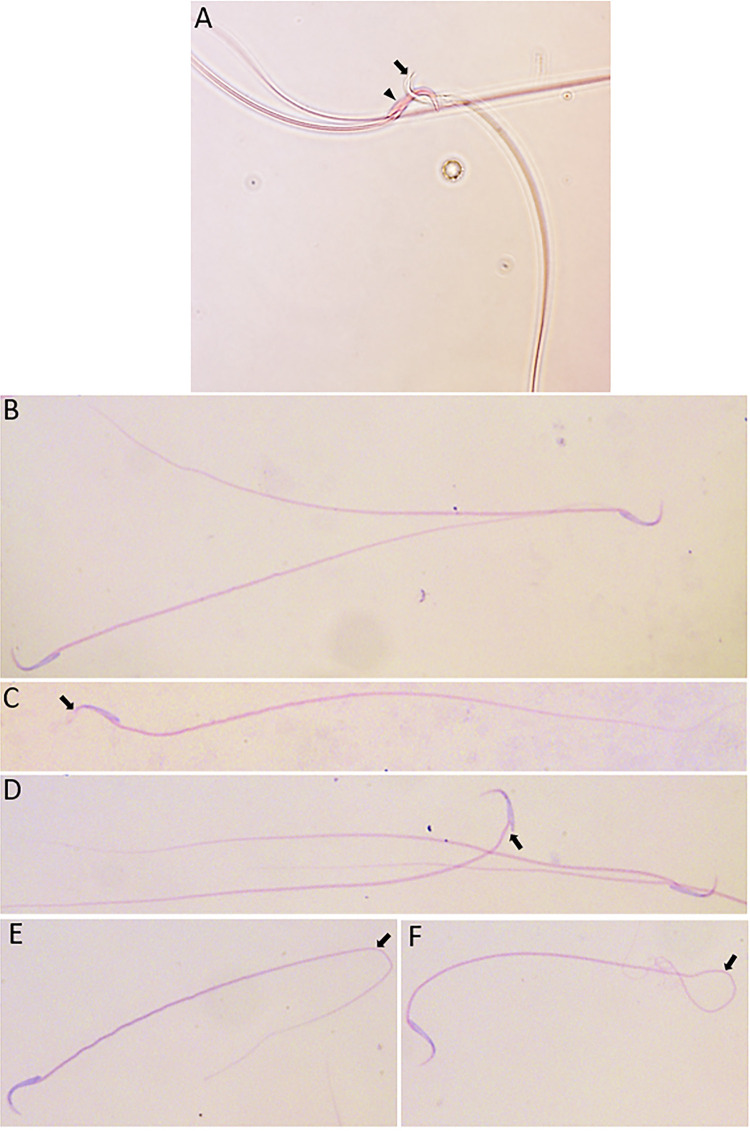
Photomicrographs of rat sperm after performing the following evaluations.

Sperm vitality was evaluated using the Eosin Y solution [modified protocol, (14)]. Fifteen microliters of the epididymal fluid and 15µL of the eosin solution were mixed on a slide and the sample was covered with a coverslip. A total of 200 spermatozoa were analyzed randomly using an OlympusBX-51 phase contrast optical microscope (Olympus-Japan) under x1000 magnification. The spermatozoa were classified as alive (sperm-unstained) and dead (sperm-stained) (Figure-1A). Then, the percentage of sperm alive in each sample was calculated.

A) Sperm vitality by Eosin Y solution: alive sperm (arrow), and dead sperm (head arrow); B-F) Sperm morphology after dye Hematoxylin-Eosin: B) normal sperm, C) abnormal spermatozoa head (arrow), D) abnormal spermatozoa middle piece (arrow), E and F) abnormal sperm tails (arrows).

### Morphology

Sperm morphology was evaluated using the dye Hematoxylin-Eosin. Fifteen microliters of the epididymis fluid were smeared on a slide. After drying, the slide with the sample was stained with hematoxylin-eosin, fixed, and air-dried. A total of 200 spermatozoa were analyzed using an OlympusBX-51 phase optical microscope (Olympus-Japan) under x1000 magnification. The percentage of abnormal spermatozoa (head, intermediate piece, or tail) (Figures 1B-F) was obtained (15).

### Motility

Motility parameters were evaluated using the Computer Assisted Sperm Analysis system (CASA; IVOS, v.12, Hamilton Thorn Research, Beverly, MA, USA). The settings were previously described by the equipment manual. A sample of epididymis fluid (15x10^6^ sperm/mL) was added to a specific slide (Leja, Nieuw-Vennep, Netherlands) pre-heated at 37 °C. Ten fields were selected for analysis regarding total motility (%), progressive motility (%), velocity average path (µm/s-VAP), velocity straight line (µm/s-VSL), velocity curved line (µm/s-VCL), amplitude of lateral head displacement (ALH), beat cross frequency (BCF), and linearity (VSL/VCL) and rectilinearity (STR).

### Nuclear DNA integrity

**Figure 2:**
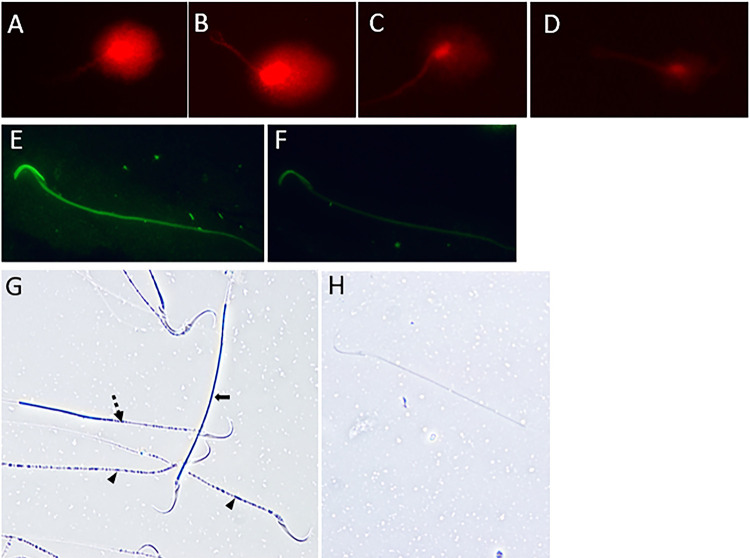
Photomicrographs of rat sperm after performing the following evaluations: A-D) nuclear DNA integrity by alkaline comet assay: A) Comet grade I (observed nucleus and no DNA migration), B) Comet grade II (observed nucleus and little DNA migration), C) Comet grade III (observed nucleus and intense DNA migration), and D) Comet grade IV (no observed nucleus and intense DNA migration); E and F) Acrosome integrity by a peanut agglutin in conjugated to a fluorescein isothiocyanate probe (PNA-FITC): E) intact acrosome, and F) non-intact acrosome; G and H) Mitochondrial activity by 3,3’-diaminobenzidine chromogen (DAB): G) Class I (100% staining - arrow), Class II (more than 50% stained - dashed arrow), Class III (less than 50% stained - head arrow), H) Class IV (without staining).

Nuclear DNA integrity was evaluated using the Alkaline Comet Assay [modified protocol, (16)]. Samples of epididymis fluid (1x10^6^ sperm/mL) were diluted in 0.75% low melting point agarose in Tris-borate-EDTA buffer (TBE, 0.089M-Tris, 0.089M-borate, and 0.002M-EDTA) at 37 ºC. One hundred microliters of diluted sperm solution were placed in a slide previously prepared with 1% normal melting point agarose in TBE in duplicate. In subsequent steps, the slides were protected from light to prevent artificial DNA damage. Slides were covered with a coverslip and kept at 4ºC for 10 minutes. Then, the coverslip was removed and 300µL of 0.75% low melting point agarose in TBE was added to the slide. It was covered with a coverslip and kept at 4°C for 10 minutes. After, the slide was immersed in two lysis buffers (100mM-Na2-EDTA, 10mM-Tris, 2.5M-NaCl, pH=11): the first with 4mM-dithiothreitol and 2% Triton X-100 at 4°C for 1 hour; and the second with 10 µg/mL proteinase K at 37ºC for 2.5 hours. The slide was washed twice in distilled water for 5 minutes. Then, the slide was immersed in an alkaline electrophoresis solution (300mM-NaOH, 1mM-Na2-EDTA, pH>13) for 20 minutes. Therefore, the electrophoresis was performed with 3 V/cm and a maximum initial current of < 400 mA, for 20 minutes. Then, the slide was washed twice in TBE buffer for 5 minutes, and subsequently, the slide was fixed in 70% and 100% ethanol. After drying, the slide was stained with SYBR-Green II-RNA gel stain (diluted 1:10,000 in TBE) for 40 minutes. The slide was washed with TBE to remove background staining, and sperm were evaluated using an OlympusBX51 epifluorescence microscope (Olympus-Japan) equipped with a rhodamine/TRITC filter under 400× magnification. One hundred sperm were scored according to the intensity of DNA damage observed by the Comet tail and nuclear intensity: I (observed nucleus and no DNA migration), II (observed nucleus and little DNA migration), III (observed nucleus and intense DNA migration), and IV (no observed nucleus and intense DNA migration) (Figure 2A-D). Then, the percentage of each grade was calculated in each sample.

### Acrosome integrity

Acrosome integrity was evaluated using a peanut agglutin in conjugated to a fluorescein isothiocyanate probe (PNA-FITC, Sigma, USA) that binds to the outer acrosomal membrane (16). For that, 15µL of epididymal fluid was smeared on the slide in duplicate, and the slide was air-dried. Then, it was fixed in 100% methanol (Merck, Brazil) for 15 minutes, and air-dried. Sperm were stained with 60 µg/mL of PNA-FITC in phosphate-buffered saline (PBS, 137mM-NaCl, 2.7mM-KCl, 10mM-Na2HPO4, and 1.8mM-KH2PO4) for 30 minutes in the dark and slide was washed with milli-Q water. A total of 200 sperm were analyzed using an OlympusBX-51 epifluorescence microscope (Olympus-Japan) equipped with a FITC filter under 1,000× magnification (Figures 2E-F). The percentage of sperm with intact acrosome was calculated in each sample.

### Mitochondrial activity

Mitochondrial activity was evaluated using the 3,3’-diaminobenzidine chromogen (DAB) (Sigma, USA) (17). The oxidation of this chromogen by the mitochondrial c oxidase results in its polymerization and deposition in the sperm middle piece. Samples of epididymal fluid and DAB solution were incubated, in which DAB solution contained 1 mg/mL. Samples were incubated in a water bath at 37 °C for 1 hour in the dark. Then, 15µL of each sample was smeared on a slide in duplicated and air-dried. Slides were fixed in 10% formaldehyde for 10 minutes and air-dried. A total of 200 spermatozoa were analyzed using an OlympusBX-51 phase contrast optical microscope (Olympus-Japan) under 1,000x magnification. Sperm were classified into 4 classes: I (middle piece completely stained), II (middle piece mostly stained), III (middle piece mostly unstained), and IV (middle piece completely unstained) (Figure 2G-H). Then, the percentage of each class was calculated in each sample.

### Statistical Analysis

Statistical analysis was performed using the SPSS18.0 software for Windows. Initially, the Kolmogorov-Smirnov test (normality test) and the Levene`s test (homogeneity test) were performed. For variables that passed through both tests, the ANOVA test followed by the Bonferroni *post-hoc* test was performed. When normality or homogeneity was not observed, the variables were transformed as z-score and the ANOVA test followed by the Bonferroni *post-hoc* test was applied. The Welch’s test was applied to obtain the *p-value*, only when the homogeneity test did not pass the variables. Variables without normal distribution and homogeneity, non-parametric Kruskal Wallis test followed by the Games-Howell *post-hoc* test was performed. An alpha of 5% was adopted.

## RESULTS

Body weight and testicular morphometrics were analyzed and there were no differences among the V40, V100, and S groups (Table-1).

**Table 1:** Body weight, testicular morphometric data, and absolute and relative weight of epididymis, ventral prostate, and seminal vesicles from rats of the Sham-control (S) and Varicocele (V40 and V100) groups.

		S (n=6)		V40 (n=6)	V100 (n=6)	*p*
**Body weight (g)**						
Mean; SD		441.83; 15.84		440.25; 55.96	445.33; 38.24	0.977
CI 95%	[425.20-458.46]		[381.52-498.98]		[405.20-485.46]	
**Testicular - IP (g)**						
Mean; SD		1.89; 0.13		1.82; 0.11	1.81; 0.07	0.383
CI 95%		[1.75-2.03]		[1.70-1.94]	[1.73-1.89]	
**Testicular – C (g)**						
Mean; SD		1.84; 0.12		1.78; 0.10	1.78; 0.07	0.514
CI 95%		[1.72-1.96]		[1.67-1.89]	[1.71-1.86]	
**Relative testicular weight - IP**						
**(**g/100 g of body weight)						
Mean; SD		0.43; 0.04		0.41; 0.06	0.42; 0.05	0.876
CI 95%		[0.39-0.47]		[0.35-0.47]	[0.37-0.47]	
**Relative testicular weight - C**						
(g/100 g of body weight)						
Mean; SD		0.42; 0.03		0.40; 0.05	0.37; 0.06	0.315
CI 95%		[0.38-0.45]		[0.35-0.46]	[0.31-0.43]	
**Testicular volume - IP (cm^3^)**						
Mean; SD		1.79; 0.13		1.72; 0.10	1.71; 0.06	0.371
CI 95%		[1.65-1.93]		[1.61-1.83]	[1.65-1.78]	
**Testicular volume - C (cm^3^)**						
Mean; SD		1.72; 0.13		1.68; 0.09	1.71; 0.09	0.821
CI 95%		[1.59-1.86]		[1.59-1.78]	[1.62-1.80]	
**Epididymis - IP (g)**						
Mean; SD		0.83; 0.05		0.81; 0.04	0.78; 0.04	0.244
CI 95%		[0.77-0.89]		[0.77-0.85]	[0.74-0.83]	
**Epididymis - C (g)**						
Mean; SD		0.84; 0.07		0.86; 0.07	0.77; 0.04	0.068
CI 95%		[0.77-0.92]		[0.79-0.93]	[0.73-0.82]	
**Relative epididymis weight - IP** (g/100g of body weight)						
Mean; SD		0.19; 0.01		0.19; 0.03	0.18; 0.02	0.457
CI 95%		[0.17-0.20]		[0.16-0.22]	[0.16-0.19]	
**Relative epididymis weight - C** (g/100g of body weight)						
Mean; SD		0.19; 0.02		0.20; 0.03	0.17; 0.02	0.080
CI 95%		[0.17-0.21]		[0.17-0.23]	[0.15-0.19]	
Ventral prostate (g)						
Mean; SD		0.44; 0.04		0.46; 0.02	0.42; 0.06	0.404
CI 95%		[0.39-0.49]		[0.43-0.48]	[0.36-0.48]	
**Relative ventral prostate weight** (g/100g of body weight)						
Mean; SD		0.10; 0.01		0.11; 0.02	0.09; 0.01	0.218
CI 95%		[0.09-0.11]		[0.09-0.13]	[0.07-0.10]	
**A pair of full seminal vesicles (g)**						
Mean; SD		1.35; 0.14^a^		1.56; 0.02^b^	1.41; 0.11^ab^	0.011*
CI 95%		[1.20-1.51]		[1.54-1.58]	[1.29-1.53]	
**Relative weight of a pair of full seminal vesicles** (g/100g of body weight)						
Mean; SD		0.31; 0.03^a^		0.38; 0.05^b^	0.31; 0.05^ab^	0.027*
CI 95%		[0.27-0.34]		[0.32-0.43]	[0.26-0.36]	
**A pair of empty seminal vesicles (g)**						
Mean; SD		0.57; 0.07^a^		0.59; 0.06^ab^	0.67; 0.05^b^	0.021*
CI 95%		[0.50-0.64]		[0.53-0.65]	[0.62-0.73]	
**Relative weight of a pair of empty seminal vesicles** (g/100g of body weight)						
Mean; SD		0.13; 0.01		0.14; 0.02	0.15; 0.02	0.255
CI 95%		[0.11-0.14]		[0.12-0.16]	[0.12-0.17]	

Standard deviation (SD); 95% confidence interval of the mean (CI 95%); grams (g); cubic centimeters (cm3) ipsilateral (IP); contralateral (C) *Statistically significant difference (p < 0.05). Different superscript letters in the same line indicate a significant difference, while the same letters in the same line indicate that a difference was not found.

Other morphometric parameters are shown in Table-1. Epididymis and prostate data showed no difference among the V40, V100, and S groups. The V40 group showed an increase in absolute (p=0.011) and relative (p=0.027) weight of a pair of full seminal vesicles when compared to the S group. On the other hand, the V100 group showed an increase in absolute weight (p=0.021) of a pair of empty seminal vesicles when compared to the S group.

The varicocele groups showed a decrease in vitality when compared to the S group, considering sperm collected from both the ipsilateral (p<0.0001) and contralateral (p<0.0001) epididymis (Table-2).

**Table 2:** Sperm vitality, sperm morphology, sperm motility, and sperm function analysis: DNA integrity, acrosome integrity, and mitochondrial activity obtained from rats of the Sham-control (S) and Varicocele (V40 and V100) groups.

	S (n=6)	V40 (n=6)	V100 (n=6)	*p*
**Vitality - IP (%)**				
Mean; SD	75.50; 4.50^a^	45.00; 7.77^b^	49.00; 13.33^b^	<0.0001*
CI 95%	[70.77-80.23]	[36.84-53.16]	[35.01-62.98]	
**Vitality - C (%)**				
Mean; SD	75.50; 4.50^a^	34.08; 7.39^b^	45.50; 16.98^b^	<0.0001*
CI 95%	[70.77-80.23]	[26.33-41.84]	[27.68-63.32]	
**Total abnormal morphology – IP (%)**				
Mean; SD	8.50; 5.42^a^	26.58; 4.52^b^	25.00; 7.29^b^	<0.0001*
CI 95%	[2.81-14.19]	[21.84-31.33]	[17.33-32.65]	
**Total abnormal morphology – C (%)**				
Mean; SD	8.58; 5.40^a^	24.00; 4.82^b^	33.17; 4.53^c^	<0.0001*
CI 95%	[2.92-14.23]	[18.94-29.05]	[28.41-37.93]	
**Total motility - IP**				
Mean; SD	45.33; 10.19^a^	30.33; 5.01^b^	26.00; 7.69^b^	0.002*
CI 95%	[34.63-56.03]	[25.08-35.59]	[17.92-34.07]	
**Total motility - C**				
Mean; SD	45.33; 10.19^a^	31.67; 5.61^ab^	25.00; 11.08^b^	0.006*
CI 95%	[34.64-56.03]	[25.78-37.55]	[13.37-36.63]	
**Progressive motility - IP**				
Mean; SD	9.33; 3.67^a^	4.00; 1.67^b^	5.33; 1.63^b^	0.006*
CI 95%	[5.48-13.18]	[2.24-5.76]	[3.62-7.05]	
**Progressive motility - C**				
Mean; SD	9.33; 3.67	5.17; 2.86	7.17; 3.43	0.130
CI 95%	[5.48-13.18]	[2.17-8.17]	[3.57-10.77]	
**DNA integrity (%) -IP**				
**Comet grade I – IP**				
Median; IR	40.25; 30.50^a^	23.00; 18.00^a^	6.50; 17.50^b^	0.005*
Q1-Q3	22.50-53.00	18.87-36.87	1.50-19.00	
**Comet grade II – IP**				
Median; IR	55.75; 31.00	51.75; 25.00	73.50; 32.00	0.100
Q1-Q3	45.75-76.75	37.50-62.50	57.50-89.50	
**Comet grade III – IP**				
Median; IR	1.50; 3.60^a^	14.00; 9.80^b^	10.50; 14.50^b^	0.005*
Q1-Q3	0.37-4.00	7.50-17.25	8.75-23.25	
**Comet grade IV - IP**				
Median; IR	0.00; 0.10^a^	7.00; 16.10^ab^	3.50; 5.50^b^	0.002*
Q1-Q3	0.00-0.12	2.87-19.00	1.75-7.25	
**DNA integrity (%) - C**				
**Comet grade I – C**				
Median; IR	40.25; 30.50^a^	16.50; 25.90^b^	20.00; 21.30^b^	0.04*
Q1-Q3	22.50-53.00	7.12-33.00	5.25-26.50	
**Comet grade II – C**				
Median; IR	55.75; 31.00	54.50; 25.30	65.50; 15.00	0.263
Q1-Q3	45.75-76.75	43.12-68.37	58.75-73.75	
**Comet grade III – C**				
Median; IR	1.50; 3.60^a^	14.25; 6.10^b^	14.50; 11.80^b^	0.004*
Q1-Q3	0.37-4.00	13.87-20.00	5.75-17.50	
**Comet grade IV - C**				
Median; IR	0.00; 0.10^a^	11.25; 8.50^b^	3.00; 6.00^a^	0.002*
Q1-Q3	0.00-0.12	6.25-14.75	0.00-6.00	
**Acrosome integrity (%) - IP**				
Mean; SD	82.33; 12.86	84.17; 2.56	81.33; 5.39	0.833
CI 95%	[68.83-95.83]	[81.48-86.86]	[75.67-86.99]	
**Acrosome integrity (%) - C**				
Mean; SD	79.67; 11.77	81.33; 2.06	83.83; 7.25	0.674
CI 95%	[67.31-92.02]	[79.17-83.50]	[76.22-91.44]	
**Mitochondrial activity (%) - IP**				
Class I – IP				
Median; IR	44.50; 33.50^a^	0.00; 1.30^b^	1.00; 2.30^b^	0.002*
Q1-Q3	20.00-53.50	0.00-1.25	1.00-2.25	
Class II – IP				
Median; IR	55.50; 30.30	5.00; 70.80	48.00; 41.50	0.291
Q1-Q3	46.50-76.75	2.75-73.50	22.75-64.25	
Class III – IP				
Median; IR	0.00; 1.80^a^	95.00; 72.00^b^	50.00; 42.80^b^	0.002*
Q1-Q3	0.00-1.75	25.25-97.25	34.50-77.25	
Class IV - IP				
Median; IR	0.00; 0.00	0.00; 0.00	0.00; 0.00	1
Q1-Q3	0.00-0.00	0.00-0.00	0.00-0.00	
Class I – C				
Median; IR	44.50; 33.50^a^	0.50; 1.80^b^	0.00; 2.00^b^	0.002*
Q1-Q3	20.00-53.50	0.00-1.75	0.00-2.00	
Class II – C				
Median; IR	55.50; 30.30^a^	5.50; 15.50^b^	34.00; 47.50^ab^	0.007*
Q1-Q3	46.50-76.75	3.50-19.00	21.00-68.50	
Class III – C				
Median; IR	0.00; 1.80^a^	94.00; 17.80^b^	66.00; 49.50^b^	<0.0001*
Q1-Q3	0.00-1.75	78.00-95.75	29.50-79.00	
Class IV - C				
Median; IR	0.00; 0.00	0.00; 0.00	0.00-0.00	1
Q1-Q3	0.00-0.00	0.00-0.00	0.00-0.00	

Standard deviation (SD); 95% confidence interval of the mean (CI 95%); interquartile range (IR); first and third quartile (Q1-Q3); ipsilateral (IP); contralateral (C) *Statistically significant difference (*p* < 0.05). Different superscript letters in the same line indicate a significant difference, while the same letters in the same line indicate that a difference was not found.

In the total abnormal morphology, the varicocele groups showed an increase when compared to the S group, considering sperm collected from both the ipsilateral (p<0.0001) and contralateral (p<0.0001) epididymis; however, the V100 group showed an increase in the contralateral when compared to V40 group (Table-2). Other analyzed parameters are in Table-3.

**Table 3:** Complementary results of sperm morphology obtained from rats of the Sham-control (S) and Varicocele (V40 and V100) groups.

	S (n=6)	V40 (n=6)	V100 (n=6)	p
**Head abnormal morphology (%) – IP**				
Mean; SD	4.50; 5.00^a^	16.17; 5.64^b^	12.17; 7.65^ab^	0.016*
CI 95%	[0.00-9.75]	[10.25-22.08]	[4.13-20.20]	
**Head abnormal morphology (%) – C**				
Mean; SD	4.50; 5.00^a^	13.25; 5.93^ab^	18.67; 7.17^b^	0.004*
CI 95%	[0.00-9.75]	[7.03-19.47]	[11.14-26.19]	
**Middle piece abnormal morphology (%) – IP**				
Mean; SD	0.33; 0.60	2.08; 2.80	2.83; 2.93	0.205
CI 95%	[0.00-0.97]	[0.00-5.02]	[0.00-5.90]	
**Middle piece abnormal morphology (%) – C**				
Mean; SD	0.33; 0.60^a^	2.42; 1.80^ab^	4.17; 2.99^b^	0.018*
CI 95%	0.00-0.97	0.53-4.31	1.02-7.31	
**Tail abnormal morphology (%) – IP**				
Mean; SD	3.75; 2.73^a^	8.33; 3.98^ab^	10.00; 4.10^b^	0.026*
CI 95%	[0.88-6.62]	[4.15-12.51]	[5.70-14.30]	
**Tail abnormal morphology (%) – C**				
Mean; SD	3.75; 2.73	8.33; 5.05	10.33; 5.00	0.055
CI 95%	[0.88-6.62]	[3.04-13.63]	[5.08-15.59]	

Standard deviation (SD); 95% confidence interval of the mean (CI 95%); ipsilateral (IP); contralateral (C); *Statistically significant difference (*p* < 0.05). Different superscript letters in the same line indicate significant differences, while the same letters in the same line indicate that a difference was not found.

Total sperm motility showed a decrease in Varicocele groups when compared to the S group, considering sperm collected from the ipsilateral epididymis (p=0.002). In the contralateral spermatozoa, this parameter also decreased when the V100 group was compared to the S group (p=0.006). Regarding the progressive motility, both varicocele groups showed a decrease compared to the S group; however, these results were significant just in the ipsilateral (p=0.006) (Table-2). Other analyzed parameters are shown in Supplementary Table-1.

The V40 and V100 groups showed worse results in DNA integrity when compared to the S group. The varicocele groups were compared; however, we did not observe a conclusive outcome. When the spermatozoa collected from the ipsilateral epididymis were analyzed, we observed an increase in spermatozoa without fragmentation (Comet grade I, p=0.005) in the V40 group when compared to the V100 group. On the other hand, spermatozoa collected from the contralateral epididymis showed an increase in spermatozoa with high fragmentation (Comet grade IV, p=0.002) in the V40 group when compared to the V100 group (Table-2).

Acrosome integrity showed no difference between groups considering sperm collected from both epididymis, ipsilateral, and contralateral (Table-2).

The varicocele groups showed worse results in mitochondrial activity when compared to the S group. There was no difference between varicocele groups (Table-2).

## DISCUSSION

The effect of the establishment time of varicocele is difficult to study in humans; therefore, rats with induced varicocele have been used as a study model (11). There are benefits to using this model such as decreased environmental co-factors and bias when compared to humans, and the possibility to analyze the testicular tissue (10, 18). Nevertheless, studies report an ipsilateral effect and a contralateral effect of the varicocele (19), leading to damage to the development of spermatogenesis, and secretory function of the Sertoli and Leydig cells on contralateral testis (20). Furthermore, ipsilateral and contralateral damage have been observed in sperm function parameters such as nuclear DNA integrity, mitochondrial activity, and acrosome integrity (21).

Varicocele could harm the reproductive organs observed in the epididymis and the testis of rats. Studies have shown harm to the histopathological and morphometrics parameters in the epididymis of adult rats with induced varicocele (22); and, a decrease in testicular weight and harm to the histopathological in rats with induced varicocele in the peripubertal phase (20).

In rats, the seminal vesicle’s secretion has relevant functions, such as the support of sperm survival after ejaculation, and the modulation of the female reproductive tract to improve embryo receptivity (23). It also acts on preimplantation to provide optimal conditions for embryo development (24). Besides that, this accessory gland may also be exposed to the physiological alterations caused by varicocele. Seminal vesicles showed altered with major weight in varicocele groups as full as empty when varicocele animals were compared to Sham-control animals. Probably, these hypertrophies could be caused by the inflammatory state, which happens as a defense cell infiltration in the inflamed organs (25). These results have been observed in the literature, that showed in rats with varicocele induced, the presence of inflammatory markers, such as interleukins (12).

Regarding sperm vitality and morphology, the varicocele groups showed worse results when compared to the Sham-control group. These results are compatible with the literature, which demonstrates that induction of experimental varicocele can induce a sperm quality alteration, with decreased sperm vitality (26) and/or alterations in sperm morphology (27). Sperm motility of varicocele groups showed a decrease in total motility and progressive sperm displacement when compared to the Sham-control group; the effect of varicocele on sperm motility showed worse values, as also observed in the literature (27).

These modifications aforementioned induced by the varicocele may happen due to physiological changes, such as the oxidative stress state generated by the excessive amount of Reactive Oxygen Species (ROS) not followed by the increase levels (28) that interfere in seminal quality by changes in the sperm cell membrane, decreasing the sperm vitality and motility by the loss of cell fluidity (29). Moreover, the varicocele alters the apoptotic process, allowing defective cells to remain in the ejaculated sample, and worsening the sperm’s normal morphology (30). Finally, the retrograde blood flow and blood stasis observed in the pathophysiology of varicocele harm the mechanism of scrotal temperature regulation. Under normal conditions, arterial blood flow is directed to the scrotum; it is cooled by the venous blood flow from the pampiniform plexus blood flow; however, in the presence of varicose veins, this system is impaired. One possible consequence of this high scrotal temperature is the loss of Sertoli cell function damaging spermatogenesis (20).

All these pathophysiological mechanisms generated by the varicocele can also affect sperm function. Sperm analysis in the current study showed alteration of the mitochondrial activity and the DNA integrity in both varicocele groups. Oxidative stress can lead to alterations in mitochondrial activity as demonstrated by the DAB results and this fact could explain to the lack of sperm motility in these animals (31). Moreover, Dieament et al. (2017) indicated that men with varicocele have an alteration in mitochondrial membrane potential leading to the inactivation of this organelle (32). Besides that, oxidative stress can modify the cellular membrane through a process known as lipid peroxidation, which also has the potential of harming the proteins and genetic material, leading to sperm DNA damage (33). In agreement with these aspects, studies indicated that sperm from a man with varicocele frequently pass by mistakes in the chromatin compaction or by abortive apoptosis process that culminates in samples with worse sperm morphology, and DNA fragmentation (34).

Aiming to verify if the duration of experimental varicocele brings worse results to sperm quality, we compared different periods of induced varicocele (120 or 60 days). Although data on the duration of experimental varicocele showed significant differences, in general, it does not seem to interfere with most of the studied parameters. The results are in accordance with Andrade-Rocha, who did not observe statistical differences in seminal quality among adolescents, adults, and older men (35). However, when Chehval and Purcell investigated varicocele men with normal semen analysis and compared samples of the same man after 8 years, they observed a decrease in sperm concentration and motility; these results may have occurred by environmental factors’ effects on men. Toxic substances, there are in food, water, air, and others, may compromise the quality of life, and consequently, sperm quality (36).

Varicocele might alter the male reproductive homeostasis by the processes mentioned above, such as oxidative stress, an increase in scrotal temperature, testicular hypoxia, and loss of blood flux (37); and, the permanence of this condition makes these consequences continue to cause sperm damage (5) independent of the established time of varicocele.

### STUDY LIMITATION

Although studies with animals require smaller groups when compared to human studies, our study worked with a few animals. Maybe the difference observed would be more evident with more numbers per group.

## CONCLUSIONS

Although both varicocele groups showed worse results compared to the Sham-control group, the establishment time of varicocele does not interfere with the biometry of reproductive organs, vitality, motility, acrosome integrity, and mitochondrial activity. With this, in the present study in rats, we conclude that varicocele acts negatively on sperm, especially on its function. On the other hand, the negative progressive effect was not observed on sperm.

## APPENDIX

Supplementary Table 1 - Complementary results of sperm motility obtained from rats of the Sham-control (S) and Varicocele (V40 and V100) groups.


