# Supplementary material for: Moment of induction and duration of experimental varicocele in rats: effects on semen quality
Source: Int Braz J Urol. 2024 Apr 25;50(4):433–49. doi: 10.1590/S1677-5538.IBJU.2023.0412 (PMC11262715; doi:10.1590/S1677-5538.IBJU.2023.0412)
Supplement: Supplementary file 1 [file 1677-6119-ibju-50-04-0433-suppl01.pdf]

APPENDIX

Supplementary Table 1 - Complementary results of sperm motility obtained from rats of the Sham-control (S) and Varicocele (V40 and V100) groups.

	S (n=6)	V40 (n=6)	V100 (n=6)	*p*
**VAP - IP**				
Mean; SD	112.10; 12.80[a]	93.83; 7.33[b]	102.82; 12.49[ab]	0.040*
CI 95%	[98.67-125.53]	[86.14-101.53]	[89.71-115.93]	
**VAP - C**				
Mean; SD	112.10; 12.80	98.22; 14.54	113.25; 20.89	0.244
CI 95%	[98.67-125.53]	[82.96-113.47]	[91.33-135.17]	
**VSL - IP**				
Mean; SD	72.23; 7.86[a]	59.18; 4.45[b]	69.88; 9.65[ab]	0.022*
CI 95%	[63.99-80.48]	[54.51-63.85]	[59.75-80.01]	
**VSL - C**				
Mean; SD	72.23; 7.86	62.60; 11.41	78.02; 14.97	0.105
CI 95%	[63.99-80.48]	[50.62-74.58]	[62.31-93.72]	
**VCL - IP**				
Mean; SD	212.02; 29.02[a]	184.55; 14.11[ab]	167.28; 19.72[b]	0.010*
CI 95%	[181.56-242.47]	[169.74-199.36]	[146.58-187.98]	
**VCL - C**				
Mean; SD	212.02; 29.02	195.03; 30.49	186.10; 29.43	0.334
CI 95%	[181.56-242.47]	[163.03-227.03]	[155.21-216.99]	
**ALH - IP**				
Mean; SD	8.78; 0.81[ab]	9.63; 1.74[a]	7.58; 0.93[b]	0.036*
CI 95%	[7.93-9.63]	[7.81-11.46]	[6.60-8.56]	
**ALH - C**				
Mean; SD	8.78; 0.81	11.82; 3.20	7.82; 3.96	0.084
CI 95%	[7.93-9.63]	[8.45-15.18]	[3.65-11.98]	
**BCF -IP**				
Mean; SD	13.48; 2.95	17.68; 2.41	13.32; 4.52	0.072
CI 95%	[10.38-16.58]	[15.15-20.21]	[8.57-18.07]	
**BCF -C**				
Mean; SD	13.48; 2.95[a]	19.23; 2.63[b]	13.58; 4.80[a]	0.021*
CI 95%	[10.38-16.58]	[16.48-21.99]	[8.55-18.62]	

**STR - IP**				
Mean; SD	64.50; 4.85	63.00; 1.79	67.50; 5.36	0.215
CI 95%	[59.41-69.59]	[61.12-64.88]	[61.88-73.12]	
**STR - C**				
Mean; SD	64.50; 4.85	64.67; 3.83	67.67; 5.12	0.432
CI 95%	[59.41-69.59]	[60.65-68.69]	[62.29-73.04]	
**LIN - IP**				
Mean; SD	35.83; 3.54[a]	32.50; 2.43[a]	43.00; 6.39[b]	0.013*
CI 95%	[32.11-39.55]	[29.95-35.05]	[36.30-49.70]	
**LIN -C**				
Mean; SD	35.83; 3.54[ab]	33.17; 4.21[a]	43.33; 8.98[b]	0.029*
CI 95%	[32.11-39.55]	[28.74-37.59]	[33.91-52.76]	

Standard deviation (SD); 95% confidence interval of the mean (CI 95%); ipsilateral (IP); contralateral (C); velocity average path (VAP); velocity straight line (VSL); velocity curved line (VCL); amplitude of lateral head displacement (ALH); beat cross frequency (BCF); linearity (LIN). *Statistically significant difference ($p < 0.05$). Different superscript letters in the same line indicate significant differences, while the same letters in the same line indicate that a difference was not found.
